# Supplementary material for: Association between Gout and Dyslipidemia: A Nested Case–Control Study Using a National Health Screening Cohort
Source: J Pers Med. 2022 Apr 8;12(4):605. doi: 10.3390/jpm12040605 (PMC9032264; doi:10.3390/jpm12040605)
Supplement: Supplementary file 1 [file jpm-12-00605-s001.zip › jpm-1655497-supplementary.pdf]

Table S1. Subgroup analyses of odds ratios (95% confidence interval) for gout in the dyslipidemia group compared with the non-dyslipidemia group according to obesity, smoking, alcohol consumption, total cholesterol, blood pressure, and fasting blood glucose.

Characteristics	Odds Ratios for Gout					
	Crude †	*p*-Value	Partial ‡	*p*-Value	Full §	*p*-Value
Obesity						
Underweight (n = 1,885)						
Dyslipidemia	2.09 (1.46–2.99)	<0.001 *	1.88 (1.25–2.84)	0.003 *	1.89 (1.25–2.86)	0.003 *
Non-dyslipidemia	1		1		1	
Normal weight (n = 27,517)						
Dyslipidemia	1.54 (1.42–1.66)	<0.001 *	1.46 (1.34–1.60)	<0.001 *	1.45 (1.33–1.58)	<0.001 *
Non-dyslipidemia	1		1		1	
Overweight (n = 23,266)						
Dyslipidemia	1.42 (1.32–1.52)	<0.001 *	1.41 (1.30–1.53)	<0.001 *	1.40 (1.29–1.52)	<0.001 *
Non-dyslipidemia	1		1		1	
Obese (n = 30,727)						
Dyslipidemia	1.47 (1.39–1.55)	<0.001 *	1.42 (1.33–1.51)	<0.001 *	1.41 (1.32–1.50)	<0.001 *

Non-dyslipidemia	1		1		1	
Smoking						
Nonsmoker (n = 46,976)						
Dyslipidemia	1.55 (1.47–1.63)	<0.001 *	1.44 (1.36–1.52)	<0.001 *	1.39 (1.31–1.47)	<0.001 *
Non-dyslipidemia	1		1		1	
Past smoker or current smoker (n = 36,419)						
Dyslipidemia	1.64 (1.55–1.74)	<0.001 *	1.57 (1.47–1.68)	<0.001 *	1.48 (1.38–1.58)	<0.001 *
Non-dyslipidemia	1		1		1	
Alcohol consumption						
<1 time a week (n = 46,476)						
Dyslipidemia	1.61 (1.53–1.69)	<0.001 *	1.45 (1.37–1.54)	<0.001 *	1.40 (1.31–1.48)	<0.001 *
Non-dyslipidemia	1		1		1	
≥1 time a week (n = 36,919)						
Dyslipidemia	1.54 (1.45–1.62)	<0.001 *	1.53 (1.44–1.63)	<0.001 *	1.45 (1.36–1.54)	<0.001 *
Non-dyslipidemia	1		1		1	
Total cholesterol (mg/dL)						

<200 (n = 46,006)						
Dyslipidemia	1.61 (1.52–1.69)	<0.001 *	1.54 (1.45–1.64)	<0.001 *	1.44 (1.35–1.54)	<0.001 *
Non-dyslipidemia	1		1		1	
≥200 to <240 (n = 26,768)						
Dyslipidemia	1.55 (1.45–1.66)	<0.001 *	1.49 (1.38–1.60)	<0.001 *	1.42 (1.32–1.53)	<0.001 *
Non-dyslipidemia	1		1		1	
≥240 (n = 10,621)						
Dyslipidemia	1.44 (1.31–1.58)	<0.001 *	1.38 (1.24–1.53)	<0.001 *	1.35 (1.21–1.49)	<0.001 *
Non-dyslipidemia	1		1		1	
Blood pressure (mmHg)						
SBP < 140 and DBP < 90 (n = 60,003)						
Dyslipidemia	1.61 (1.54–1.68)	<0.001 *	1.50 (1.43–1.58)	<0.001 *	1.42 (1.35–1.50)	<0.001 *
Non-dyslipidemia	1		1		1	
SBP ≥ 140 or DBP ≥ 90 (n = 23,392)						
Dyslipidemia	1.55 (1.45–1.65)	<0.001 *	1.47 (1.36–1.58)	<0.001 *	1.42 (1.31–1.53)	<0.001 *
Non-dyslipidemia	1		1		1	

Fasting blood glucose (mg/dL)						
<100 (n = 49,715)						
Dyslipidemia	1.73 (1.65–1.82)	<0.001 *	1.58 (1.49–1.67)	<0.001 *	1.50 (1.42–1.59)	<0.001 *
Non-dyslipidemia	1		1		1	
≥100 (n = 33,680)						
Dyslipidemia	1.41 (1.33–1.49)	<0.001 *	1.37 (1.29–1.46)	<0.001 *	1.31 (1.23–1.40)	<0.001 *
Non-dyslipidemia	1		1		1	

Abbreviations: CCI, Charlson comorbidity index; DBP, diastolic blood pressure; SBP, systolic blood pressure. * Logistic regression, Significance at $p < 0.05$. † Crude model: Adjusted for age, sex, income, and region of residence. ‡ Partially adjusted model: adjusted for age, sex, income, region of residence, the days of statin used, total cholesterol, SBP, DBP, and fasting blood glucose. § Fully adjusted model: Adjusted for age, sex, income, and region of residence, the days of statin used, total cholesterol, SBP, DBP, fasting blood glucose, obesity, smoking, alcohol consumption, and CCI scores (Subgroup analyses according to obesity, smoking, and alcohol consumption did not include obesity, smoking, and alcohol consumption as a covariate, respectively).

Table S2. Odds ratios (95% confidence interval) for gout based on the days of statin used per 1 year with subgroup analyses according to age and sex.

Characteristics	Odds Ratios for Gout					
	Crude [†]	*p*-Value	Partial [†‡]	*p*-Value	Full [†§]	*p*-Value
Total participants (n = 83,395)						
Statin used (per 1 year)	1.29 (1.24–1.33)	<0.001 *	1.11 (1.07–1.15)	<0.001 *	1.07 (1.03–1.11)	0.001 *
Age < 60 years old, men (n = 34,370)						
Statin used (per 1 year)	1.36 (1.27–1.45)	<0.001 *	1.10 (1.02–1.18)	0.018 *	1.04 (0.97–1.13)	0.281
Age < 60 years old, women (n = 7,680)						
Statin used (per 1 year)	1.43 (1.25–1.64)	<0.001 *	1.18 (1.01–1.38)	0.036 *	1.15 (0.99–1.35)	0.077
Age ≥ 60 years old, men (n = 32,020)						
Statin used (per 1 year)	1.22 (1.16–1.28)	<0.001 *	1.07 (1.02–1.13)	0.010 *	1.04 (0.98–1.10)	0.178
Age ≥ 60 years old, women (n = 9,325)						
Statin used (per 1 year)	1.33 (1.23–1.43)	<0.001 *	1.23 (1.12–1.34)	<0.001 *	1.20 (1.09–1.31)	<0.001 *

Abbreviations: CCI, Charlson comorbidity index; DBP, diastolic blood pressure; SBP, systolic blood pressure. * Conditional logistic regression, Significance at $p < 0.05$. [†] Models were stratified by age, sex, income, and region of residence. [‡] Partially adjusted model: Adjusted for dyslipidemia history, total cholesterol, SBP, DBP, and fasting blood glucose. [§] Fully adjusted model: Adjusted for dyslipidemia history, total cholesterol, SBP, DBP, fasting blood glucose, obesity, smoking, alcohol consumption, and CCI scores.
